# Overcoming Oxidative Stress in Parkinson’s Disease: NADPH Oxidase 4 (NOX4) as a Potential Therapeutic Target

**DOI:** 10.3390/antiox15050571

**Published:** 2026-05-01

**Authors:** Xinyi Xu, Qicheng Wang, Ziqi Liu, Jing Li, Sainan Wang, Li Qian

**Affiliations:** 1 The First School of Clinical Medicine, Faculty of Medicine, Yangzhou University, Yangzhou 225009, China; yxx20260521@163.com (X.X.); qihuaizi1@163.com (Q.W.); 2 Key Laboratory of the Jiangsu Higher Education Institutions for Integrated Traditional Chinese and Western Medicine in Senile Diseases Control, Yangzhou University, Yangzhou 225009, Chinam17851665090@163.com (S.W.)

**Keywords:** Parkinson’s disease, NADPH oxidase 4 (NOX4), oxidative stress, therapeutic strategies

## Abstract

Parkinson’s disease (PD) lacks effective disease-modifying therapies (DMTs). While oxidative stress drives PD pathogenesis, broad-spectrum antioxidants frequently fail in clinical trials due to limited specificity and poor cerebral bioavailability. In PD, reactive oxygen species (ROS) arise from multiple intracellular sources, among which mitochondrial dysfunction is widely recognized as a fundamental driver, while nicotinamide adenine dinucleotide phosphate (NADPH) oxidase 4 (NOX4), a constitutively active NOX isoform that predominantly generates hydrogen peroxide (H_2_O_2_), has emerged as an important enzymatic contributor in the central nervous system. This review systematically examines the important role of NOX4 in PD and proposes a mechanistic framework by which NOX4-derived ROS contribute to PD progression. NOX4-derived ROS may directly promote mitochondrial dysfunction, proteostasis disruption, neuroinflammation, and ferroptosis. More importantly, NOX4-derived ROS may aggravate mitochondrial dysfunction to increase mitochondrial ROS production, thereby promoting PD progression indirectly. We systematically summarize the emerging NOX4-targeted strategies, including highly selective small-molecule inhibitors, natural products, gene therapies, and blood–brain barrier-penetrating nanodrug delivery systems. NOX4 should be viewed as an important regulator and potential amplifier that can affect multiple pathogenic processes in PD, thereby representing a promising avenue for the development of DMTs for PD.

## 1. Introduction

Parkinson’s disease (PD) has emerged as the fastest-growing neurodegenerative disorder worldwide [1,2], imposing substantial healthcare burdens on society [3,4]. Clinically, PD is characterized by motor symptoms such as resting tremor and bradykinesia, as well as increasingly recognized non-motor manifestations including cognitive impairment and sleep disturbances [5]. The pathological hallmarks of PD are the progressive loss of dopaminergic (DA) neurons in the substantia nigra pars compacta (SNpc) and the formation of Lewy bodies resulting from the abnormal aggregation of α-synuclein (α-Syn) [6,7]. Although genetic, environmental, and aging factors collectively contribute to the progression of PD [8,9,10,11], the precise etiology and pathogenesis remain incompletely understood. Current standard treatments, including levodopa (L-Dopa) replacement therapy and deep brain stimulation (DBS), primarily alleviate symptoms but fail to halt neurodegeneration [12,13,14]. Moreover, long-term treatment is frequently associated with complications such as L-Dopa-induced dyskinesia (LID) [15,16]. Therefore, the development of disease-modifying therapies (DMTs) that target the underlying mechanisms of PD is an urgent priority [12,17].

Oxidative stress is widely acknowledged as a core contributor to PD pathogenesis [18,19,20,21,22]. DA neurons are particularly vulnerable to oxidative stress due to the substantial generation of reactive oxygen species (ROS) during dopamine metabolism [23,24]. In PD, dysregulated iron metabolism, α-Syn misfolding, and mitochondrial dysfunction further exacerbate oxidative stress, ultimately leading to the irreversible neuronal damage and death [19,25,26]. Importantly, ROS also play a crucial role in signal transduction under physiological conditions [27]. However, traditional antioxidants that indiscriminately scavenge ROS have failed to demonstrate significant neuroprotective effects [28,29,30]. These findings underscore the need for therapeutic approaches that selectively target specific endogenous sources of ROS.

Among the various endogenous sources of ROS [31,32,33,34,35], mitochondria are recognized as principal contributors, generating mitochondrial ROS (mtROS) through electron leakage from the electron transport chain (ETC), complex I deficiency, mitochondrial DNA (mtDNA) damage, and defective energy metabolism [36,37,38]. Beyond mtROS, the nicotinamide adenine dinucleotide phosphate (NADPH) oxidase (NOX) family has garnered significant attention as a major enzymatic source [39]. Increasing evidence indicates that NADPH oxidase 4 (NOX4) plays an important role in PD pathogenesis due to its unique catalytic activity, distinct subcellular localization, and widespread expression in the central nervous system (CNS) [40,41,42]. On the one hand, NOX4 participates in pathological processes including proteostasis disruption, neuroinflammation, and ferroptosis [41,42]; on the other hand, it exacerbates inherent mitochondrial dysfunction, creating a vicious cycle with mitochondrially generated ROS to indirectly advance PD processes.

Given the emerging role of NOX4 in PD, targeting NOX4 may offer new opportunities for the development of DMTs. This review systematically summarizes the molecular structure of NOX4 and discusses its contributions to PD pathogenesis, while highlighting emerging therapeutic strategies, including small-molecule inhibitors, natural product derivatives, gene therapies, and nanodrug delivery systems (NDDSs). Compared with conventional antioxidants approaches, targeting NOX4 enables selective suppression of pathological ROS generation while preserving physiological oxidative signaling. Despite significant challenges in clinical translation, a deeper understanding of NOX4-associated mechanisms in PD and the development of more effective targeting strategies may facilitate new perspectives for precise therapies, thereby accelerating the research and development of novel DMTs. Before delving into the specific pathogenic mechanisms and therapeutic targeting of NOX4 in PD, the following section provides a brief, foundational overview of the NOX family to contextualize the unique catalytic profile and functional significance of NOX4.

## 2. NOX: Biology and CNS Relevance

### 2.1. Molecular Structure, Classification, and Functions of NOX

**Figure 1:**
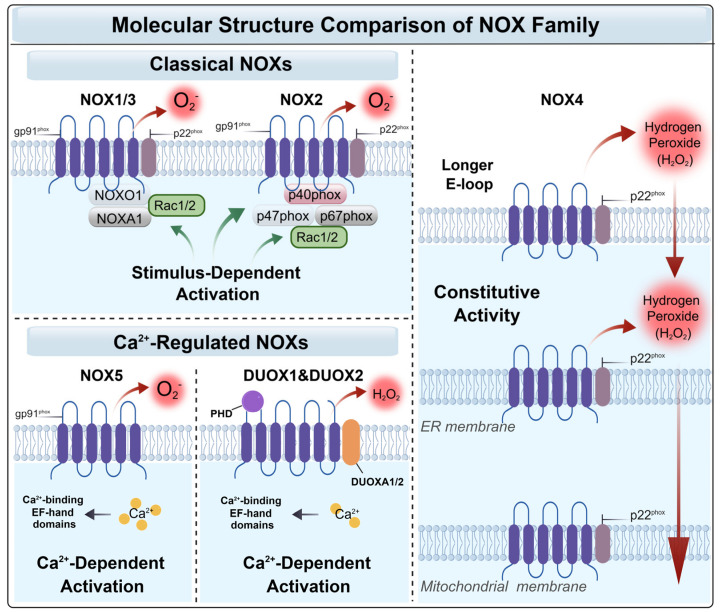
Molecular Structure of NADPH Oxidases (NOXs) in the CNS. The figure delineates three distinct subgroups of the NOX family and their respective activation mechanisms. Created with BioGDP.com and accessed on 29 April 2026 [53]. Classical NOXs (NOX1-3): Activity is contingent upon the assembly of cytosolic subunits (e.g., p47phox, Rac1), with superoxide anion (O_2_^•−^) serving as the predominant product. Unlike NOX2, which requires the cytosolic regulatory subunits p47phox and p67phox for assembly and activation, the assembly of NOX1 and NOX3 requires the homologous cytosolic factors NOXO1 and NOXA1 to substitute for these two subunits. NOX4: Predominantly localized to the endoplasmic reticulum (ER) and mitochondrial membranes, this isoform exhibits unique constitutive activity. Attributable to its distinct E-loop architecture, NOX4 facilitates the direct liberation of hydrogen peroxide (H_2_O_2_). Calcium-regulated NOXs (NOX5, DUOX1/2): Characterized by the presence of EF-hands, their enzymatic activity is modulated by intracellular Ca^2+^ concentrations. NOX5 is directly activated by four EF-hands. The DUOX family containing two EF-hands and a PHD (peroxidase-homology domain) necessitates association with DUOXA1/2 maturation factors, primarily generating H_2_O_2_. Green arrows indicate the recruitment of cytosolic subunits. Red arrows on the left indicates the chemical reactions that generate H_2_O_2_ or O_2_^−^. The long red arrow on the right indicates that NOX4 can produce H_2_O_2_ on the ER and mitochondrial membranes.

The NOX family comprises membrane-bound enzymes with seven isoforms: NOX1, NOX2, NOX3, NOX4, NOX5, DUOX1, and DUOX2. Their primary function is to catalyze the oxidation of NADPH, reducing molecular oxygen to superoxide anions (O_2_^•−^), thereby generating one of the endogenous sources of intracellular ROS [43,44]. A detailed comparison of the molecular structures and activation mechanisms governing the NOX family is summarized in Figure 1.

Most NOX isoforms require complex assembly for activation. For instance, the prototype NOX2 (gp91phox), along with NOX1 and NOX3, relies on the stabilization protein p22phox and the recruitment of various cytosolic regulatory subunits upon stimulation [43,45,46,47,48,49]. Conversely, NOX5 and DUOX1/2 are activated by intracellular calcium binding rather than cytosolic subunit assembly [35,43,47,50,51].

In contrast, NOX4 exhibits distinct structural and regulatory characteristics. Although it requires interaction with p22phox for stability, its activation does not depend on the recruitment of cytosolic subunits. Instead, NOX4 displays constitutive activity and continuously generates ROS in the absence of specific stimuli [48,52]. Notably, NOX4 predominantly produces H_2_O_2_ rather than O_2_^•−^ [52]. Studies indicate that the third extracellular loop (E-loop) of NOX4 contains an additional 28 amino acids compared with other isoforms, which may impede the release of O_2_^•−^ and promote its rapid dismutation into H_2_O_2_ [35,52]. This unique catalytic profile enables NOX4 to play a specific role in maintaining cellular redox homeostasis and mediating oxidative stress signaling.

The remaining NOX family members—NOX5 and DUOX1/2—represent an alternative activation modality. Their activation is independent of p22phox and instead mediated by intracellular calcium-binding to EF-hand domains [35,47]. Specifically, NOX5 has four EF-hand calcium-binding domains at its N-terminus. Increased cytosolic Ca^2+^ concentrations induce conformational changes that relieve autoinhibition and directly activate enzymatic activity [50]. DUOX1/2 are the most structurally complex members. In addition to two intracellular EF-hand calcium-binding domains, they possess a unique extracellular peroxidase-homology domain (PHD) that enables them to generate H_2_O_2_ and utilize it for downstream oxidative reactions [43]. However, their functional maturation requires interaction with specific accessory factors, DUOXA1/2 [51].

Overall, NOX family enzymes regulate numerous physiological processes, including cell signaling, immune defense, cell growth, and differentiation, by generating distinct types of ROS. However, dysregulated NOX activity leads to excessive ROS production and oxidative stress resulting in cellular damage. This is particularly relevant in the nervous system, which is highly sensitive to oxidative stress and closely linked to the progression of neurodegenerative diseases (NDDs).

### 2.2. CNS Distribution of NOX and Neurodegenerative Diseases

**Figure 2:**
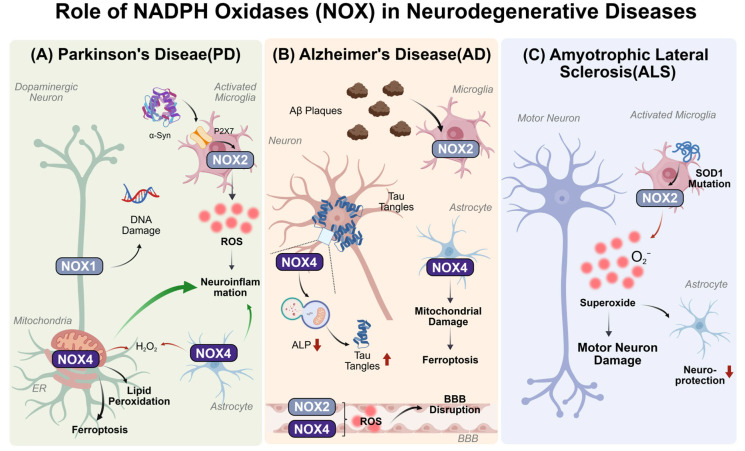
Role of NADPH Oxidases (NOX) In Neurodegenerative Diseases. This figure illustrates the pathogenic mechanisms of the NOX family across three neurodegenerative diseases (NDDs). Created with BioGDP.com and accessed on 29 April 2026 [53]. PD: NOX1 in dopaminergic (DA) neurons induces DNA damage; α-synuclein (α-Syn) activates microglial NOX2 to trigger neuroinflammation; NOX4 in DA neurons exacerbates injury by inducing ferroptosis while concurrently participating in the modulation of neuroinflammation. AD: amyloid-β (Aβ) activates microglial NOX2 to generate reactive oxygen species (ROS); NOX4 promotes neuronal Tau protein accumulation and impairs astrocytic mitochondria; additionally, both isoforms collectively disrupt the blood–brain barrier (BBB). Amyotrophic Lateral Sclerosis (ALS): Superoxide dismutase 1 (SOD1) mutations persistently activate microglial NOX2, releasing O_2_^•−^ that directly damages motor neurons and diminishes the neuroprotective efficacy of astrocytes. Red arrows indicate the chemical reactions that generate ROS, H_2_O_2_ or O_2_^−^. Green arrows indicate the promoting effect on pathological changes.

Members of the NOX family are widely expressed in various cell types within the CNS, including neurons, astrocytes, and microglia [54,55,56,57]. As the main source of endogenous ROS, the abnormal NOX activation contributes to oxidative stress, neuroinflammation, mitochondrial dysfunction, and aberrant protein aggregation, constituting a common pathological foundation for NDDs such as Alzheimer’s disease (AD), Parkinson’s disease (PD), and Amyotrophic Lateral Sclerosis (ALS) [57,58,59,60,61]. The roles of distinct NOX isoforms (particularly NOX1, NOX2, and NOX4) in driving these pathological events across different CNS cell types in various NDDs are illustrated in Figure 2.

In the context of PD, different NOX isoforms contribute to the pathogenesis through distinct cellular localizations and mechanistic pathways [57]. For example, NOX1 expression is primarily upregulated in DA neurons of the SNpc, where it contributes to oxidative DNA damage [62]. Conversely, NOX2, predominantly expressed in microglia, is activated by α-Syn and serves as a potent driver of neuroinflammation [63,64,65]. Notably, NOX4 may contribute to PD pathology through mechanisms distinct from those of other NOX isoforms. Unlike other isoforms that generate O_2_^•−^, NOX4 predominantly produces H_2_O_2_ and localizes within the endoplasmic reticulum (ER), nucleus, and mitochondria of neurons, astrocytes, endothelial cells, and pericytes [41,66,67,68,69]. Previous studies have demonstrated that NOX4 overexpression in DA neurons and astrocytes is associated with lipid peroxidation, ferroptosis, and inflammatory amplification [41,42,70]. Collectively, the current evidence supports NOX4 as an important contributor to PD progression and as a potential therapeutic target.

In AD, NOX2 primarily mediates amyloid-β (Aβ)-induced microglial activation and vascular injury [71,72], whereas NOX4 directly promotes pathological Tau protein accumulation by disrupting the autophagy-lysosomal pathway (ALP) and induces ferroptosis in astrocytes [73,74,75]. In ALS, NOX2 serves as a major contributor to motor neuron degeneration, with its activation closely associated with superoxide dismutase 1 (SOD1) mutations and the dysregulation of purinergic P2X7 receptors, among other related factors [39,76,77,78,79,80].

Additionally, NOX3 is primarily associated with cerebellar and inner ear functions [81,82,83,84], NOX5 has been implicated in blood–brain barrier (BBB) disruption [85,86,87], and DUOX1/2 expression has rarely been reported in the human brain. Given the distinct distribution and functional specificity of NOX isoforms in the CNS, the development of isoform-specific therapeutic strategies represents an important direction for future research.

## 3. The Role of NOX4-Mediated Oxidative Stress in PD

**Figure 3:**
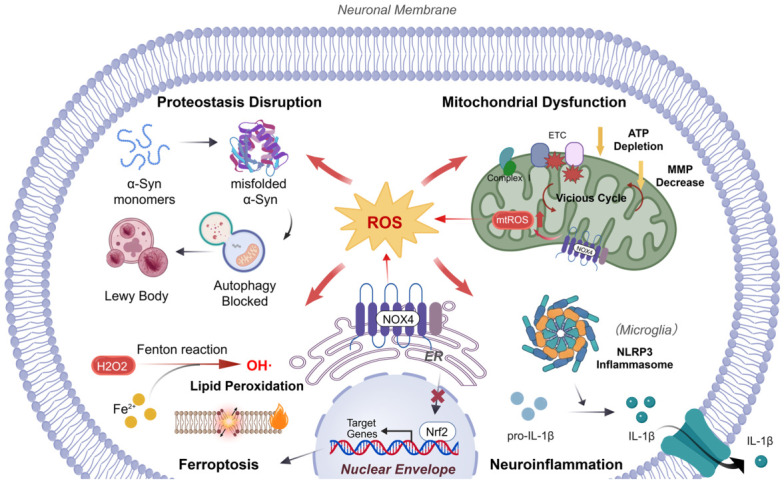
NOX4-related Pathological Mechanisms in Parkinson’s Disease. This figure shows that the upregulation of NOX4, particularly at the ER and mitochondrial membranes, contributes to intracellular H_2_O_2_ generation and may amplify a deleterious network of signaling pathways. Created with BioGDP.com and accessed on 27 April 2026 [53]. Proteostasis Disruption: Elevated oxidative stress impairs autophagy-lysosomal pathway (ALP), promoting the accumulation of misfolded α-Syn monomers into toxic aggregates and eventual Lewy body formation. Mitochondrial Dysfunction: NOX4-derived H_2_O_2_ may exacerbate mitochondrial oxidative injury, contributing to loss of mitochondrial membrane potential, reduced ATP production, and enhanced mtROS. Neuroinflammation: ROS accumulation triggers the assembly of the NLRP3 inflammasome in microglia, catalyzing the cleavage of pro-IL-1β into mature IL-1β, thereby exacerbating the neuroinflammatory environment. Ferroptosis: In the presence of Fe^2+^, H_2_O_2_ undergoes the Fenton reaction to generate highly reactive ^•^OH, driving lipid peroxidation. The sustained activation of NOX4 is often associated with the inhibition of Nrf2 signaling, thereby promoting ferroptosis.

Although oxidative stress is widely recognized as a major driver of DA neuronal degeneration in PD, the recurrent failure of traditional broad-spectrum antioxidant therapies in clinical trials underscores the need for more precise targeting of specific ROS sources [57,88]. In PD, oxidative stress is viewed as a multi-source process. Dysfunctional mitochondria are widely regarded as a dominant source of ROS, particularly through ETC disruption, defective energy metabolism, and impaired mitochondrial quality control [89,90,91]. NOX4 has attracted increasing attention owing to its constitutive activity, subcellular localization, and ability to generate H_2_O_2_ [48,57,70]. On the one hand, NOX4 participates in pathological processes including proteostasis disruption, neuroinflammation, and ferroptosis. Even more notably, NOX4-derived ROS exacerbates inherent mitochondrial dysfunction and promotes further mtROS generation, acting synergistically with the amplified mtROS to accelerate PD-related pathological progression. Therefore, NOX4 plays an important role in exacerbating the pathological progression of PD. NOX4-related core pathological mechanisms in PD are summarized in Figure 3.

### 3.1. NOX4 and Mitochondrial Dysfunction

Mitochondrial dysfunction is considered one of the key contributors to DA neuronal degeneration in PD [20]. In addition to their central role in ATP generation, mitochondria regulate numerous cellular processes, including metabolic signaling, calcium homeostasis, ROS generation, and apoptosis [92]. Defects in ETC activity, impaired ATP production, mtDNA damage, abnormal calcium handling, dopamine oxidation, and defective mitophagy can independently increase mtROS levels and drive neuronal vulnerability [36,37,38]. Increasing evidence suggests that NOX4-derived ROS may aggravate mitochondrial dysfunction, which accelerates PD progression by disrupting energy metabolism, interfering with the regulation of metabolic enzymes, and affecting mitochondrial quality control.

In certain cellular contexts, NOX4 has been reported to localize to mitochondrial membrane, and its overexpression may threaten mitochondrial energy metabolism. The H_2_O_2_ produced by NOX4 can oxidatively damage mitochondrial membrane lipids, leading to increased membrane permeability and loss of membrane potential, thus compromising ATP synthesis. Studies in glial cells have further demonstrated that abnormal NOX4 upregulation suppresses the levels of certain protein complexes in the mitochondrial ETC, thereby directly impairing mitochondrial respiratory function [74].

Recent studies have further identified an interaction between NOX4 and mitochondrial metabolic enzymes, and the dysregulation of this interaction contributes to oxidative damage in PD. In rotenone-induced PD models, short-chain enoyl-CoA hydratase 1 (ECHS1), an essential mitochondrial metabolic enzyme, directly binds to NOX4 and inhibits its activation. Under the pathological conditions, however, this regulatory mechanism is impaired, leading to uncontrolled NOX4 activity and elevated mtROS production [93]. Restoring the inhibitory effect of ECHS1 on NOX4 significantly reduces oxidative damage and alleviates DA neuronal loss.

Beyond direct damage, NOX4-mediated oxidative stress can also indirectly affect mitochondrial quality control mechanisms such as mitochondrial dynamics and mitophagy. Regarding dynamics, NOX4 abnormally regulates dynamin-related protein 1 (Drp1) activity through ROS-dependent pathways, potentially promoting excessive mitochondrial fission and generating dysfunctional fragments that are prone to apoptosis [94]. In addition, excessive ROS may also interfere with mitophagy by oxidatively modifying key proteins in the PINK1/Parkin pathway, thereby impairing the recognition and clearance of damaged mitochondria [95]. The accumulation of damaged mitochondria further amplifies intracellularly ROS production and exacerbates oxidative stress.

Furthermore, mitochondrial dysfunction exacerbated by NOX4 may also affect other key pathological processes in PD. Specifically, excessive mtROS accumulation can compromise intracellular protein degradation systems, thereby disrupting proteostasis. Additionally, mtROS can promote glial cell activation to amplify neuroinflammation, and increase cellular susceptibility to lipid peroxidation and ferroptosis. Notably, throughout these pathological events, NOX4-mediated ROS and mitochondria-generated mtROS do not exist in isolation; their potential synergy may further advance the cellular pathological evolution.

### 3.2. NOX4 and Proteostasis Disruption

Proteostasis is a fundamental cellular process that maintains proper folding, assembly, localization, and degradation of proteins, thereby ensuring neuronal health and function [88]. Its disruption, particularly the misfolding and aberrant aggregation of α-Syn, is a key pathological hallmark of PD [89,90]. While NOX4-related oxidative stress plays an important role in driving this pathological cascade, it is increasingly recognized that oxidative stress in PD arises from convergent ROS sources, rather than from NOX4 alone. In particular, mtROS together with NOX4-derived H_2_O_2_, likely acts synergistically to promote oxidative protein modifications, destabilize α-Syn, and facilitate its misfolding and aggregation [68,91,92,93]. In addition, oxidatively modified α-Syn can interact with microglial surface integrin CD11b, which further amplifies neuroinflammation [94].

NOX4-mediated oxidative stress also exacerbates proteostasis disruption by impairing intracellular protein degradation pathways. Under physiological conditions, misfolded or damaged proteins are removed through the autophagy-lysosomal pathway (ALP) and the ubiquitin-proteasome system (UPS). However, both systems are functionally compromised in PD [95,96]. Importantly, the UPS is highly ATP-dependent [96,97]. Mitochondrial dysfunction not only generates excessive mtROS but also leads to severe ATP depletion, which fundamentally impairs UPS-mediated protein clearance. In NDDs, NOX4 has been identified as a significant contributor to autophagy impairment. Building on this mitochondria-driven vulnerability, ROS generated by NOX4 disrupt ALP integrity and hinder the clearance of pathological proteins, whereas NOX4 inhibition restores autophagic activity [72]. Given that α-Syn clearance is highly dependent on functional autophagy, this mechanism is particularly relevant to PD pathogenesis.

Notably, a bidirectional regulatory mechanism exists between NOX4 stability and autophagy. Hypoxia has been shown to inhibit ALP, resulting in intracellular accumulation of NOX4 protein and excessive H_2_O_2_, thereby exacerbating oxidative stress [97]. Given the widespread prevalence of localized hypoxic microenvironments in PD pathology, a similar mechanism may occur in DA neurons. Additional evidence supporting the central role of NOX4 in proteostasis regulation shows that exosome-derived miR-100-5p modulates the NOX4/ROS/Nrf2 pathway by suppressing the upstream NOX4 expression, thereby reducing oxidative damage and improving impaired protein degradation [39].

In summary, alongside the oxidative modification of α-Syn and impairment of autophagic clearance, NOX4-related oxidative stress further contributes to proteostasis disruption in PD by exacerbating mitochondrial dysfunction, thereby compromising the function of protein degradation systems. Therefore, targeting NOX4 to alleviate oxidative stress may represent a promising and effective strategy to restore proteostasis in patients with PD.

### 3.3. NOX4 and Neuroinflammation

Neuroinflammation is one of the key pathological features of PD and is characterized by activation of glial cells, which release pro-inflammatory cytokines, chemokines, and ROS that contribute to DA neuronal damage [98,99,100]. Throughout this progress, NOX4 plays an important role by regulating glial cells activation and amplifying inflammatory signaling pathways.

Microglia are the primary mediators of neuroinflammation in the CNS. Although NOX2 is the primary source of ROS during the respiratory burst in microglia, the sustained ROS production mediated by NOX4 during chronic inflammation is critically important. Studies have revealed that lipopolysaccharide (LPS) stimulation upregulates NOX4 expression in microglia, leading to increased ROS production and pro-inflammatory cytokines secretion such as IL-6 and IL-8, ultimately reducing neuronal viability [101]. Given that peripheral infections and LPS exposure can exacerbate neuroinflammation in PD, NOX4 may represent a crucial regulatory factor in the cross-system pathological transmission process.

Astrocytes also undergo reactive astrogliosis in PD, and NOX4 is a potential contributor in the transition of astrocytes toward the neurotoxic A1 phenotype. For example, activating transcription factor 3 (ATF3) can induce NOX4 expression in DA neurons, which subsequently upregulates the pro-inflammatory mediator lipocalin-2 (LCN2) in astrocytes, recruiting and activating additional microglia to amplify the inflammatory cascade [41].

Elevated NOX4 expression not only contributes to astrocytic dysfunction [70], but also affects neighboring neuronal survival through paracrine signaling. He et al. demonstrated that mesenchymal stem cell-derived exosomes carrying miR-100-5p can directly target and inhibit NOX4, restoring the ROS-Nrf2 signaling balance, and significantly alleviating neuroinflammation and DA neuronal damage [42].

Furthermore, NOX4 has been implicated in activation of the NLRP3 inflammasome. ROS generated by NOX4 can induce the dysregulation of NLRP3/NLRP6 inflammasomes, thereby enhancing the maturation and release of inflammatory cytokines such as IL-1β [102]. Considering that the hyperactivation of the NLRP3 inflammasome is a well-established mechanism in PD [103], NOX4 likely exerts an important regulatory role in this progress.

Overall, NOX4-mediated oxidative stress synergistically promotes neuroinflammation in PD by regulating glial cell activation, activating pro-inflammatory pathways, and inducing NLRP3 inflammasome activation. Targeting NOX4 to reduce oxidative stress and neuroinflammation holds promise as a modifying strategy for PD.

### 3.4. NOX4 and Ferroptosis

Ferroptosis is an iron-dependent form of regulated cell death characterized by excessive accumulation of lipid peroxides, and it has emerged as a critical contributor to DA neuronal loss in PD [104]. The substantia nigra of the brain contains high levels of iron ions, and DA neurons are highly susceptible to oxidative stress and mitochondrial dysfunction, making this region extremely vulnerable to ferroptosis [105]. As an important source of H_2_O_2_, NOX4 provides the substrate for the Fenton reaction and acts as an emerging factor in initiating and accelerating ferroptosis.

A recent study has shown that NOX4 directly promotes ferroptosis in PD through the ATF3/PKCα signaling axis. In MPTP-induced PD mouse models, ATF3 upregulates NOX4 expression in DA neurons and astrocytes, triggering lipid peroxidation and abnormal iron accumulation in the SNpc. Furthermore, the interaction between NOX4 and activated protein kinase Cα (PKCα) further heightens cellular susceptibility to ferroptosis [41].

In addition to promoting oxidative damage, NOX4 also suppresses the Nrf2/GPX4 antioxidant defense pathway. GPX4 is a key inhibitor of ferroptosis and is regulated by transcription factor Nrf2 [106,107]. Under pathological conditions in PD, the sustained NOX4 activation is frequently accompanied by inhibition of the Nrf2 signaling. Pharmacological studies support this mechanism: both avicularin and astragalosides have been shown to modulate the NOX4/Nrf2 signaling axis, suppress NOX4, promote nuclear translocation of Nrf2, and restore GPX4 expression and cerebral iron homeostasis, thereby alleviating ferroptosis [108,109].

Collectively, NOX4 promotes ferroptosis by enhancing oxidative stress and repressing endogenous antioxidant defenses. Therefore, targeting NOX4 and its downstream signaling cascades represents a therapeutic potential for slowing PD progression.

## 4. Therapeutic Strategies Targeting NOX4

Given the emerging role of NOX4-mediated oxidative stress in the pathogenesis of PD, particularly its involvement in multiple pathological cascades including proteostasis disruption, mitochondrial dysfunction, neuroinflammation, and ferroptosis, targeting NOX4 is likely to be combinatorial rather than sufficient as monotherapy for the development of DMTs. Unlike conventional broad-spectrum ROS scavengers, selective NOX4 inhibitors suppress the excessive generation of disease-associated ROS at an enzymatic source, thus mitigating oxidative damage while potentially preserving physiological redox signaling [39,55].

**Figure 4:**
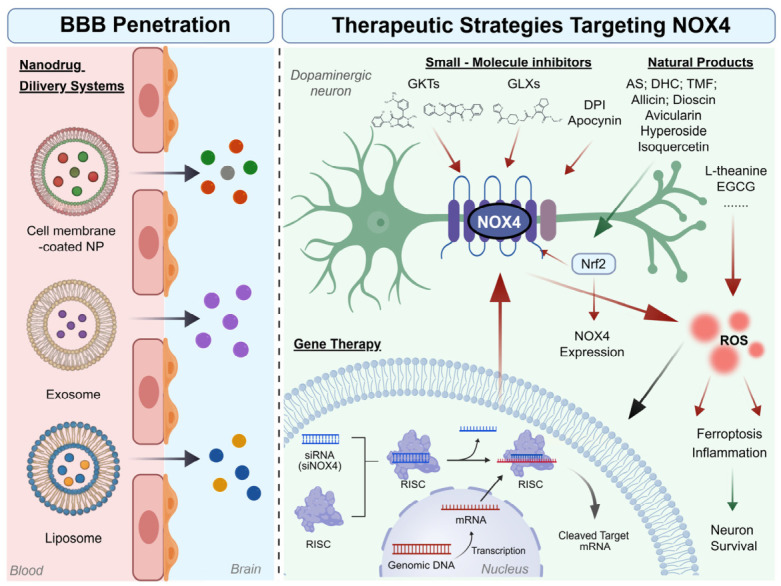
Multidimensional Therapeutic Strategies Targeting NOX4 for PD Intervention. The figure illustrates comprehensive approaches to inhibit NOX4 activity and expression in DA neurons, overcoming the BBB for precise neuroprotection. Created with BioGDP.com and accessed on 25 March 2026 [53]. BBB Penetration (Left Panel): Nanocarriers, including cell membrane-coated nanoparticles (NP), exosomes, and liposomes, facilitate the delivery of therapeutic agents across the BBB. Therapeutic Mechanisms in Neurons (Right Panel): Three modalities target NOX4-related pathology: (1) Pharmacological Inhibition: Small-molecule inhibitors, ranging from specific NOX4 inhibitors (e.g., GKTs, GLXs) to broad-spectrum inhibitors (e.g., DPI, Apocynin), directly block NOX4 enzymatic activity; (2) Natural Products: Bioactive compounds modulate NOX4 expression through the Nrf2 axis (e.g., AS, DHC, TMF…) or directly scavenge ROS (e.g., L-theanine, EGCG…); (3) Gene Therapy: Small interfering RNA (siRNA) delivery triggers RNA-induced silencing complex (RISC)-mediated NOX4 gene knockdown. Red arrows indicate an inhibitory effect. The green arrows indicate a promoting effect.

Current therapeutic approaches targeting NOX4 mainly include synthetic small-molecule inhibitors, naturally derived active compounds with multi-target advantages, gene regulatory technologies, and nanodrug delivery systems (NDDSs). This section systematically summarizes these therapeutic strategies and discusses their pharmacological mechanisms and translational potential. Multidimensional therapeutic strategies targeting NOX4 for PD intervention are illustrated in Figure 4.

### 4.1. Small-Molecule Inhibitors

#### 4.1.1. Broad-Spectrum NOX Inhibitors

Early studies relied on broad-spectrum NOX inhibitors such as diphenyleneiodonium (DPI) and apocynin to explore the role of NOX enzymes in disease pathogenesis. Although these compounds lack specificity, they provided important experimental evidence supporting the causal relationship between NOX-mediated oxidative stress and NDDs such as PD.

DPI acts as a flavoprotein inhibitor that blocks electron transfer by irreversibly binding to the conserved FAD-binding domain [110,111,112,113]. It exhibits potent inhibitory activity at micromolar or even nanomolar level [114]. In experimental PD models, DPI demonstrates neuroprotective effects by suppressing microglia-mediated oxidative stress and reducing the release of pro-inflammatory cytokines and nitric oxide (NO). These effects are mediated in part through inhibition of ERK phosphorylation. DPI also attenuates persistent microglial activation during chronic inflammation, thereby protecting DA neurons in the substantia nigra [115,116]. However, the clinical application of DPI is limited by its poor specificity. DPI concurrently inhibits multiple flavoenzymes, including xanthine oxidase (XO), cytochrome P450, and nitric oxide synthase (NOS). This broad and non-specific action leads to significant cytotoxicity and systemic side effects, restricting DPI primarily to use as a pharmacological research tool [117].

Apocynin has also been widely studied as a NOX inhibitor with translational potential. However, its mechanism of action remains controversial. Traditional models suggest that apocynin inhibits NOX activity by preventing translocation of the p47phox subunit. Other studies indicate that apocynin primarily acts as a ROS (especially H_2_O_2_) scavenger and may exert a partial inhibitory effect on NOX4 [35,118,119]. In PD models, apocynin can effectively reduce microglial activation, attenuate oxidative stress, protect DA neurons, and improve motor symptoms [120,121]. Furthermore, to overcome limitations in bioavailability, several apocynin derivatives have been developed. The dimeric derivative diapocynin shows its improved BBB permeability and significantly suppresses MPTP-induced expression of the NOX gp91phox subunit, thereby reducing oxidative stress and neuroinflammation while restoring striatal dopamine levels [122]. Another derivative, mito-apocynin, is targeted to mitochondria and delays neurodegeneration in MitoPark transgenic mice by blocking NOX2 activation in microglia [123]. These findings highlight the potential drug development focusing on apocynin derivatives to enhance their pharmacological efficacy and targeting capabilities.

Although these compounds have validated the therapeutic value of NOX inhibition in PD, their limited specificity and uncertain mechanisms underscore the need for more selective NOX4-targeted therapies.

#### 4.1.2. GKT Series Inhibitors

To overcome the limitations of early broad-spectrum inhibitors, considerable efforts have focused on developing highly selective NOX inhibitors. The GKT series compounds, developed by GenKyoTex and acquired by Calliditas Therapeutics [124,125], represent some of the most extensively studied NOX inhibitors. Among these pyrazolopyridine dione derivatives, GKT137831 (setanaxib) and GKT136901 are dual NOX1/4 inhibitors with promising pharmacological properties.

• GKT137831 (Setanaxib)

GKT137831 is currently one of the most extensively characterized NOX inhibitors and exhibits high affinity for both NOX1 and NOX4, along with good oral bioavailability [126]. Early studies primarily investigated its antifibrotic properties [127], but recent research has suggested potential therapeutic applications in PD. Zhang et al. demonstrated that GKT137831 effectively alleviates PD-associated central hyperalgesia by inhibiting NOX4 activity in the periaqueductal gray matter, thereby reducing oxidative stress and restoring γ-aminobutyric acid-ergic (GABAergic) signaling pathways [128]. Moreover, damage to the neurovascular unit frequently accompanies PD progression. GKT137831 has been shown to significantly attenuate neurological dysfunction in models of cerebral ischemia–reperfusion injury and subarachnoid hemorrhage, thus preserving BBB integrity and improving cerebral microcirculation [67,129,130]. However, some studies have raised concerns regarding potential side effects. Yoshikawa et al. reported that GKT137831 may inhibit proliferation of neural stem/progenitor cells, thereby interfering with physiological neurogenesis [131]. These findings highlight the need for further investigation of its safety profile in neurodegenerative conditions.

• GKT136901

GKT136901 is a structural analog of GKT137831 and functions as an efficacious dual NOX1/4 inhibitor. Experimental studies have shown that GKT136901 reduces hippocampal neuronal death and ROS production under hypoxic conditions, while also suppressing increased permeability of human brain endothelial cells, thereby preserving BBB integrity and providing neuroprotection [132]. In AD models, GKT136901 specifically inhibits NOX4-mediated H_2_O_2_ production induced by amyloid-β40 (Aβ40) in brain endothelial cells [133]. These findings suggest that GKT136901 may effectively improve neurovascular unit dysfunction and has potential therapeutic value in NDDs such as PD.

#### 4.1.3. GLX Series Inhibitors

The GLX series inhibitors, named after the abbreviation of the company calledGlucox Biotech (Stockholm, Sweden), represent a novel generation of NOX inhibitors with high selectivity for the NOX4 isoform. These compounds were first reported by the group of Dr. Nils Welsh and developed to minimize off-target effects on other NOX enzymes, with better safety profile and more precise antioxidant therapies [134,135].

• GLX351322

GLX351322 is the most extensively studied compound in the GLX series in the context of neuroprotection. In PD-related models, Zhao et al. demonstrated that GLX351322 significantly reduces ROS production in SH-SY5Y cells by inhibiting NOX4. This compound also restores mitochondrial membrane potential (MMP) and mitochondrial complex activity, thereby reversing mitochondrial dysfunction-induced neuronal apoptosis [93]. In addition, GLX351322 has been reported to inhibit ferroptosis. Both the GLX351322 and its nanoformulations effectively reduce hippocampal neuronal ferroptosis by suppressing NOX4 expression, activating the Nrf2/HO-1/GPX4 antioxidant pathway, and decreasing lipid peroxidation and iron accumulation [136,137]. Given that ferroptosis is one of the key mechanisms underlying nigral neuronal loss in PD, GLX351322 represents a promising candidate for DMTs of PD. Moreover, its broad-spectrum anti-inflammatory capabilities across various disease models further support its translational potentials [102,138,139,140].

• GLX7013114

GLX7013114 is another novel NOX4 inhibitor within the GLX series that has attracted considerable attention. Research on this compound has largely focused on the neurovascular unit, which plays an important role in ameliorating microcirculatory disturbances in PD brain. Studies using retinal models, commonly considered as extensions of CNS neurovascular research, have shown that GLX7013114 protects neurons by reducing caspase-3 activation, suppressing microglia-mediated neuroinflammation and reducing blood–retinal barrier leakage [141,142]. Additionally, GLX7013114 has demonstrated protective effects in other organs, including maintenance of mitochondrial homeostasis and inhibition of fibrosis in kidney and lens models respectively [143,144]. These properties suggest its potential therapeutic value in preventing glial scar formation and mitochondrial failure during PD progression, making it a promising multi-target disease-modifying candidate.

### 4.2. Natural Product Derivatives

Natural products and their derivatives have attracted significant attention for the treatment of NDDs because of their multi-target mechanisms, relatively low toxicity, and diverse chemical structures [145,146]. Although current studies specifically targeting NOX4 in PD remain limited, the shared pathological features among NDDs, such as oxidative stress, neuroinflammation, and ferroptosis, provide important insights into potential therapeutic applications.

Centering on the core pathological mechanisms of PD, this section systematically reviews several classes of natural products, including flavonoids, alkaloids, terpenoids, saponins, phenolics, and quinones. These compounds modulate relevant pathological pathways associated with NOX4 activity and therefore may have therapeutic potential in PD. The major NOX4-targeted natural product derivatives with therapeutic potential for PD and their mechanisms are summarized in Table 1.

#### 4.2.1. Flavonoids

Flavonoids are polyphenolic compounds with potent antioxidant and anti-inflammatory properties [176]. Numerous studies have indicated that flavonoids can attenuate oxidative stress by inhibiting NOX4 activity.

Given that mitochondrial dysfunction is a key contributor to DA neuronal death in PD, safflor yellow B (SYB) has been shown to improve mitochondrial function and inhibit apoptosis by suppressing NOX4-mediated ROS generation [147]. To address neuroinflammatory responses associated with glial hyperactivation during the progression of PD, poncirin and (−)-epicatechin inhibit NLRP3 inflammasome activation and alleviate low-grade chronic inflammation through downregulation of NOX4 expression, respectively [148,149]. Flavonoids also modulate ferroptosis-related pathways. Avicularin significantly attenuates lipid peroxidation by inhibiting NOX4 and activating the Nrf2 antioxidant pathway [108]. In addition, all the aforementioned pathological alterations converge upon oxidative stress, which constitutes the key mechanism of action of flavonoids. For example, 5,6,7,4′-tetramethoxyflavanone (TMF) alleviates impairments in synaptic plasticity by activating Nrf2 [150], whereas isoquercetin promotes Nrf2 nuclear translocation and inhibit the NOX4/ROS/NF-κB pathway [151]. Other flavonoids, including hyperoside, ampelopsin (AMP), and diosmin, attenuates neuronal injury by inhibiting the expression of NOX4 or its associated subunits [152,153,154,155]. Collectively, these compounds establish a robust and multifaceted antioxidant defense by targeting NOX4-mediated oxidative stress.

However, most evidence for NOX4 modulation by flavonoids derives from models of cerebral ischemia and AD [108,147,148,150,151,153,154]. Studies directly evaluating their efficacy in PD models remain limited, and future research is required to clarify the pharmacokinetic profiles and translational potential in PD.

#### 4.2.2. Alkaloids

Alkaloids represent a structurally diverse class of natural compounds with significant neuroprotective potential. Many alkaloids modulate NOX4 to address various pathological stages of PD progression.

In the early stage of PD, BBB disruption and oxidative damage are prominent pathological events. Dihydrocapsaicin (DHC) protects the BBB integrity by suppressing NOX4 and activating the Nrf2 pathway, thereby enhancing endogenous antioxidant and anti-inflammatory responses [157]. Similarly, the tetramethylpyrazine (TMP) analog CXC195 inhibits both NOX2 and NOX4, reducing oxidative stress at its source [156]. Autophagy impairment is another important pathological feature of PD. Although primarily studied in AD models, galanthamine has been shown to stabilize neuronal autophagy by inhibiting NOX4, suggesting potential relevance for preventing the abnormal protein aggregation from autophagic dysregulation in PD [158]. In addition, oxymatrine (OMT) exerts anti-apoptotic effects by inhibiting NOX4 enzymatic activity and specifically downregulating the expression of executioner protein caspase-3, thereby directly inhibiting neuronal apoptosis [159].

Despite these promising findings, most studies on alkaloids were conducted in stroke and diabetic brain injury models, and further validation in PD-specific models is required.

#### 4.2.3. Terpenoids

Terpenoids possess favorable lipophilicity and strong BBB permeability, making them promising candidates for the treatment of NDDs. Terpenoids primarily rescue damaged neurons by modulating NOX4-mediated oxidative stress and inflammatory responses. Betulinic acid has been shown to directly inhibit NOX4 expression, reduce ROS generation, and attenuate neuronal apoptosis [160]. In addition, 6-O-angeloylplenolin (6-OAP) significantly suppresses microglial activation by downregulating the NF-κB/NOX4 axis [161].

Although current evidence is primarily derived from models of peripheral inflammation or ischemia, the strong brain-penetrating capacity of terpenoids highlights their potential as promising drug candidates for PD.

#### 4.2.4. Saponins

Saponins are characterized by multi-target profiles and low toxicity, making them attractive candidates for neuroprotective therapies.

Dioscin has been shown to suppress oxidative stress-induced neuroinflammation by inhibiting the RAGE/NOX4 pathway, while simultaneously activating the Nrf2/HO-1 antioxidant axis to reduce NF-κB-mediated inflammatory responses [162]. Ferroptosis, driven by iron deposition and lipid peroxidation, is another key pathological mechanism in PD. Astragalus membranaceus saponins (AS) reduce lipid peroxidation by inhibiting NOX4 activity while upregulating the SLC7A11/GPX4 pathway to restore iron homeostasis [109]. Additionally, ginsenoside Rb1/3 (GS-Rb1/3) protect endothelial cells and preserve BBB integrity by suppressing NOX4 activity, thereby limiting peripheral-central immune crosstalk [163,164].

While these mechanisms highlight the therapeutic potential of saponins, further studies are required to confirm their efficacy in PD-specific models.

#### 4.2.5. Phenols

Natural phenolic compounds possess flexible aromatic ring structures and strong redox activities, enabling them to precisely regulate NOX4-amplified programmed cell death and the immune microenvironment.

Mulberrofuran G (MG) suppresses ER stress by inhibiting NOX4 activity, thereby preventing neuronal apoptosis [165]. Similarly, vitexin compound B-1 (VB-1) downregulates NOX4 expression by upregulating miR-92b [166]. In addition to apoptosis, ferroptosis is increasingly recognized, driving the loss of DA neurons in PD pathology. For example, methyl ferulic acid (MFA) reduces oxidative stress and delays neuronal ferroptosis by downregulating NOX4 expression in neuropathic pain models [167]. Several phenolic compounds also modulate the neuroinflammatory microenvironment. Epigallocatechin-3-gallate (EGCG) and acteoside suppress neuroinflammation by inhibiting the TLR4-NOX4 and the NF-κB pathways, respectively [168,169]. Moreover, resveratrol maintains BBB integrity by downregulating NOX4 expression [170].

Although these findings undoubtedly demonstrate broad-spectrum neuroprotective potentials, further in-depth studies are urgently required in a PD-specific pharmacological framework.

#### 4.2.6. Quinones

Quinones are highly redox-active compounds that specifically target NOX4-mediated oxidative stress pathways in the regulation of neuronal homeostasis.

Thymoquinone (TMQ), which has been validated in AD models, inhibits the RAGE/NOX4 pathway, thereby alleviating ROS generation and lipid peroxidation [171]. These effects may also be relevant for mitigating PD-associated neurotoxicity. Another quinone compound, plumbagin, suppresses NOX4-mediated ROS generation and blocks the activation of both NLRP3 inflammasome and p38/MAPK pathway [172,173]. These mechanisms further support NOX4 as a potentially relevant therapeutic target for PD.

#### 4.2.7. Other Compounds

Beyond the aforementioned compounds, several structurally unique amino acid derivatives and organosulfur compounds have also exhibited significant neuroprotective potential in PD through modulation of NOX4-related pathways.

L-theanine, which readily crosses the BBB, reduces oxidative stress by inhibiting NOX4-mediated ROS generation and suppresses neuronal ferroptosis by upregulating GPX4 and downregulating transferrin receptors (TFRC) [136]. Moreover, sulforaphane (SFN) predominantly maintains the homeostasis of the neurovascular unit by inhibiting NOX4 activity and reducing inflammatory damage to cerebral microvascular endothelial and glial cells [130]. However, its long-term efficacy during PD progression remains unclear. Allicin improves mitochondrial dysfunction and suppresses neuroinflammation by restoring the NOX/Nrf2 imbalance and inhibiting NLRP3 inflammasome activation in metabolic- and stress-induced injury, demonstrating its dual capabilities in mitochondrial improvement and immunomodulation [174,175].

Taken together, these structurally diverse compounds modulate NOX4-mediated oxidative stress through multiple mechanisms and provide a valuable experimental foundation for the development of PD-targeted therapeutic strategies. Nevertheless, further studies are needed to validate their efficacy and safety in PD-specific model.

### 4.3. Other Therapeutic Strategies

Given that NOX4 exhibits constitutive enzymatic activity and contributes to sustained oxidative stress in NDDs, therapeutic strategies beyond small-molecule inhibitors and natural product derivatives are being explored. These include gene therapy and NDDSs, which aim to modulate NOX4 expression or activity with greater precision to achieve neuroprotection effects. The features of these different therapeutic strategies are summarized in Table 2.

#### 4.3.1. Gene Therapy

Compared to conventional pharmacology, gene therapy offers a promising approach for PD, because it can achieve long-lasting intervention and may overcome the limitations of drug delivery across the BBB. Gene therapy involves treating diseases by introducing, silencing, or editing specific genes. Current strategies primarily use small interfering RNA (siRNA) or short hairpin RNA (shRNA) to silence the *NOX4* gene [182]. Knockdown of NOX4 in neurons and vascular endothelial cells significantly reduces oxidative stress and apoptosis while improving microcirculatory dysfunction [177,178,179,180,181]. These findings provide valuable insights for treating neurovascular unit dysfunction associated with PD progression.

However, the lack of cellular selectivity remains a major limitation. NOX4 exhibits profound cellular heterogeneity: it promotes ferroptosis and neuroinflammation in neurons [41], while driving pro-inflammatory signaling in astrocytes [192]. Consequently, recent therapeutic strategies are shifting from pan-brain gene silencing towards cell-specific gene regulation using promoters such as GFAP [193]. Compared with global NOX4 knockdown, astrocyte-specific inhibition has shown greater efficacy in alleviating mitochondrial dysfunction and ferroptosis [75].

#### 4.3.2. Nanodrug Delivery Systems (NDDSs)

NDDSs use nanomaterials as carriers to deliver therapeutic agents or genetic materials to specific cells or tissues, aiming to enhance therapeutic efficacy while minimizing side effects. In the treatment of PD, NDDSs can facilitate transport of NOX4 inhibitors across the BBB and improve their bioavailability and efficacy.

Early studies predominantly used liposomes and polymeric nanoparticles for antioxidants delivery [183,184]. More recently, biomimetic carriers integrating cell membrane characteristics have emerged to further enhance targeting efficiency and circulation stability. These carriers can deliver siNOX4 to selectively suppress NOX4 expression, thereby inhibiting oxidative stress and neuronal apoptosis at the source [185,186,194]. Building upon this, exosomes, cell-secreted endogenous carriers with innate BBB-penetrating capabilities, have also emerged as promising delivery systems. They exhibit unique advantages in delivering specific microRNAs (miRNAs) or siRNAs to modulate signaling pathways and gene expression, thus enabling precise genetic-level interventions [42,187,188]. In addition, novel materials such as nanozymes, gold nanoparticles, and black phosphorus nanosheets, can mimic natural antioxidant enzyme activities and act synergistically with NOX4 inhibitors to further broaden therapeutic perspectives in PD [189,190,195].

Despite their potential in targeting NOX4 for PD therapy, NDDSs still face limitations, including complex quality assessment and uncertain long-term biosafety [188,196]. Addressing these critical issues will be essential for future clinical translation.

## 5. Clinical Translation, Opportunities, and Challenges

Targeting NOX4 for the treatment of PD offers promising opportunities for the development of DMTs. However, successful clinical translation requires overcoming several challenges spanning drug development, targeted delivery strategies, and clinical validation.

A major limitation in early NOX inhibitors is their poor isoform specificity. Because members of the NOX family share high structural homology, early inhibitors frequently interfere with the functions of other isoforms, leading to adverse reactions. Therefore, future drug design should focus on the unique structural domains of NOX4 to achieve precise targeting. Furthermore, balancing the physiological and pathological functions of NOX4 is essential. NOX4 plays an important role in maintaining cellular differentiation and redox homeostasis, and complete or prolonged inhibition may lead to adverse consequences [43]. Consequently, future studies should aim to selectively regulate the subcellular localization of NOX4 (such as within the mitochondria) or its specific activation pathways, to suppress pathological signaling while preserving its physiological functions.

Even when effective inhibitors are available, the BBB remains a major obstacle to drug delivery in PD. Fortunately, NDDSs provide promising strategies to improve BBB penetration and enhance therapeutic efficacy [183,184,189,197,198]. Biomimetic nanocarriers, exosome-based delivery systems, and exploiting the meningeal lymphatic pathway may facilitate efficient transport of therapeutic agents or genetic materials (e.g., siRNA) into the brain, thereby providing new opportunities for precision medicine [42,183,184,185,186,187,188,198]. Another important consideration is the multifactorial nature of PD pathogenesis. Disease progression involves multiple processes, including α-Syn aggregation, neuroinflammation, mitochondrial dysfunction, and ferroptosis [199]. Although mitochondrial dysfunction is a primary and well-established initiator of oxidative stress in PD, NOX4 functions as a significant enzymatic amplifier in this pathogenic network. Given this complex synergy, targeting NOX4 alone may therefore be insufficient to halt disease progression. Combining NOX4-targeted strategies with other neuroprotective approaches, such as mitochondrial protection, anti-ferroptosis therapies, or nanodrugs, to achieve multi-target synergistic treatment, may yield better therapeutic outcomes [189,197,200,201,202,203,204,205].

Ultimately, the clinical translation of NOX4-targeted therapies must address limitations in delayed clinical diagnosis and discrepancies in experimental models. Changes in NOX4 expression levels may serve as potential biomarkers for the early diagnosis of PD [40], facilitating earlier interventions and maximally improving clinical outcomes. Simultaneously, leveraging multi-omics analyses and organoid models can bridge the gap between animal models and humans. Integration of these approaches with precision medicine and patient stratification strategies will effectively improve the reliability of preclinical research and increase the success rate of clinical trials [206,207,208].

Taken together, although significant challenges remain, advances in drug design, targeted delivery technologies, and clinical validation may enable NOX4-targeted strategies to become viable therapeutic options for PD.

## 6. Conclusions

Therapeutic strategies targeting NOX4 represent a promising direction in PD drug development, moving beyond conventional broad-spectrum antioxidant approaches toward a more precise modulation of pathological ROS sources. Although mitochondrial dysfunction remains a fundamental driver of intracellular ROS, NOX4-derived ROS not only directly exacerbates the underlying mitochondrial dysfunction but also acts in concert with mitochondria-driven damage to collectively propel downstream pathological cascades, including proteostasis disruption, glial-mediated neuroinflammation, and ferroptosis. Targeting NOX4 has therefore emerged as a promising therapeutic strategy, particularly because it offers a druggable means of reducing pathological ROS generation. Highly selective small-molecule inhibitors (such as the GKT and GLX series) and natural products possessing multiple pharmacological activities have demonstrated the potential to block NOX4-related pathological mechanisms. In parallel, NDDSs incorporating exosomes and biomimetic nanocarriers, as well as gene-therapy approaches, provide innovative strategies to overcome the BBB restrictions and enable precise intracerebral delivery. Despite these advances, significant challenges remain before NOX4-targeted therapies can be translated into clinical practice. Further in-depth exploration of the structural biology and specific regulatory mechanisms of NOX4 are needed to overcome the therapeutic bottlenecks in PD and achieve disease-modifying therapies.

## Figures and Tables

**Table 1:** Natural Product Derivatives Targeting NOX4 and Their Potential Therapeutic Significance for PD.

Category	Name	Disease Model	NOX4-Related Mechanism	Potential Therapeutic Significance for PD	References
Flavonoids	Safflor yellow B, SYB	Ischemic Brain Injury	Modulate AK046177/miR-134/CREB axis; Downregulate NOX4 expression; Improve mitochondrial function and cellular respiration	Mitochondrial protection	[147]
	Poncirin	Cerebral Ischemia/Reperfusion (I/R) Injury and Hypoxia–Ischemia Encephalopathy (HIE)	Modulate NOX4/ROS/NLRP3 axis; Inhibit NOX4 expression; Attenuate neuroinflammatory responses	Alleviation of neuroinflammation	[148]
	(−)-Epicatechin	High-fat diet (HFD)-induced Obesity-related Cognitive Impairment	Inhibit NOX4 expression (Potentially associated with the suppression of metabolic endotoxemia and neuroinflammation)	Alleviation of neuroinflammation	[149]
	Avicularin	Alzheimer’s disease, AD	Modulate NOX4/Nrf2 axis; Inhibit cellular ferroptosis	Alleviation of iron metabolism dysregulation	[108]
	5,6,7,4′-Tetramethoxyflavanone, TMF	AD	Inhibit NOX4 expression; Improve Nrf2 antioxidant pathway and synaptic plasticity impairment; Inhibit cellular senescence and apoptosis	Antioxidation	[150]
	Isoquercetin	I/R Injury	Modulate Nrf2-mediated NOX4/ROS/NF-κB pathway; Inhibit NOX4 expression; Suppress neuronal apoptosis	Antioxidation	[151]
	Hyperoside	Spinal Cord Injury, SCI	Activate PI3K/AKT and Nrf2/HO-1 pathways; Inhibit expression of oxidative stress markers including NOX4; Inhibit neuroinflammation	Antioxidation; Alleviation of neuroinflammation	[152]
	Scutellarin	Cerebral I/R Injury	Target and modulate AR-NOX axis; Inhibit NOX4 expression	Antioxidation	[153]
	Ampelopsin, AMP	AD	Inhibit NOX4 expression; Attenuate neuroinflammatory and oxidative stress	Antioxidation; Alleviation of neuroinflammation	[154]
	Diosmin	Arsenic-induced Neurotoxicity	Inhibit expression of NOX4 and its subunits (gp91phox, p47phox); Suppress neurotoxicity	Antioxidation	[155]
Alkaloids	Tetramethylpyrazine, TMP	Cerebral I/R Injury	(Its analog CXC195) Inhibit NOX2/4 and iNOS expression; Exert antioxidant action	Antioxidation	[156]
	Dihydrocapsaicin, DHC	Focal Cerebral I/R Injury	Downregulate NOX4 expression; Activate Nrf2 pathway; Reduce inflammation, oxidative stress and BBB disruption; Attenuate neuronal apoptosis	Antioxidation; Alleviation of neuroinflammation; Maintenance of BBB integrity	[157]
	Galanthamine	AD	Inhibit NOX4 expression; Suppress Aβ-mediated neuronal autophagy; Attenuate neuronal apoptosis	Stabilization of autophagic function	[158]
	Oxymatrine, OMT	Diabetic Brain Injury	Inhibit NOX2/4 expression to suppress oxidative stress; Downregulate caspase-3 expression to inhibit apoptosis	Antioxidation; Anti-apoptosis	[159]
Triptolides	Betulinic Acid	Cerebral I/R Injury	Downregulate NOX4 expression; Attenuate neuronal apoptosis	Antioxidation; Anti-apoptosis	[160]
	6-O-angeloylplenolin, 6-OAP	Lipopolysaccharide(LPS)-induced Neuroinflammation	Downregulate NOX4 expression to suppress oxidative stress; Inhibit NF-κB pathway and pro-inflammatory cytokine expression to suppress neuroinflammation	Antioxidation; Alleviation of neuroinflammation	[161]
Saponins	Dioscin	AD	Downregulate RAGE/NOX4 pathway; Upregulate Nrf2/HO-1 antioxidant pathway; Inhibit NF-κB/AP-1 inflammatory pathway; Attenuate oxidative stress and neuroinflammatory	Antioxidation; Alleviation of neuroinflammation	[162]
	Astragalus membranaceus saponins, AS	AD	Modulate NOX4/Nrf2 pathway; Upregulate SLC7A11/GPX4 pathway; Attenuate lipid peroxidation and ferroptosis	Alleviation of iron metabolism dysregulation	[109]
	Ginsenoside Rb1, GS-Rb1	Cerebral Ischemia	Inhibit NOX4 expression; Attenuate neuroinflammation; Protect BBB integrity	Alleviation of neuroinflammation; Maintenance of BBB integrity	[163]
	Ginsenoside Rg3, GS-Rb3	Multiple Sclerosis, MS	Inhibit NOX2/4 expression; Protect BBB integrity; Suppress neuroinflammatory and spinal cord demyelination	Alleviation of neuroinflammation; Maintenance of BBB integrity	[164]
Phenols	Mulberrofuran G, MG	Cerebral Ischemia	Inhibit NOX4 expression; Suppress ER stress; Attenuate neuronal death	Antioxidation	[165]
	Vitexin compound B-1, VB-1	Cerebral I/R Injury	Modulate miR-92b/NOX4 pathway; Inhibit NOX4 expression; Attenuate neuronal apoptosis	Anti-apoptosis	[166]
	Methyl ferulic acid, MFA	Neuropathic Pain	Inhibit NOX4 expression; Exert antioxidant effects; Suppress neuronal ferroptosis	Antioxidation; Alleviation of iron metabolism dysregulation	[167]
	Epigallocatechin-3-gallate, EGCG	X-irradiation-induced Cognitive Impairments	Inhibit TLR4-NOX4 pathway in microglia; Suppress NF-κB-mediated neuroinflammation	Alleviation of neuroinflammation	[168]
	Acteoside	Cerebral I/R Injury	Inhibit NOX4 expression; Attenuate oxidative stress; Modulate NF-κB signaling to attenuate inflammation	Antioxidation; Alleviation of neuroinflammation	[169]
	Resveratrol	MS	Inhibit NOX4 expression; Protect BBB integrity; Attenuate oxidative stress and neuroinflammation	Antioxidation; Alleviation of neuroinflammation; Maintenance of BBB integrity	[170]
Quinones	Thymoquinone, TMQ	AD	Downregulate Aβ-induced RAGE/NOX4 pathway; Attenuate oxidative stress and neuronal pathology	Antioxidation	[171]
	Plumbagin	Cerebral I/R Injury [172]; Traumatic Brain Injury, TBI [173]	Inhibit NOX4 expression; Suppress NLRP3 inflammasome activation and NF-κB pathway; Attenuate neuroinflammation and apoptosis [172]. Modulate NOX4/ROS/p38 MAPK axes; Inhibits NOX4 expression; Attenuate neuronal apoptosis [173].	Alleviation of neuroinflammation [172]; Anti-apoptosis [172,173]	[172,173]
Others	L-theanine	Sleep Deprivation, SD	Inhibit NOX4 expression; Suppress oxidative stress and ferroptosis	Antioxidation; Alleviation of iron metabolism dysregulation	[136]
	Sulforaphane, SFN	inflammation and excitotoxicity	Inhibit NOX4 expression; Exert antioxidant and anti-apoptotic effects	Maintenance of neurovascular unit homeostasis	[130]
	Allicin	HFD-induced depressive-like behaviors [174]; chronic social defeat stress (CSDS)induced depressive-like behaviors [175]	Inhibit NOX4 expression; Correct NOX/Nrf2 imbalance; Modulate mitochondrial function and autophagy [174]. Inhibit NOX4 expression; Upregulate Nrf2/HO-1 antioxidant pathway; Suppress NLRP3 inflammasome; Attenuate neuroinflammation and neuronal apoptosis [175].	Antioxidation; Stabilization of autophagic function; Mitochondrial protection [174]; Alleviation of neuroinflammation; Anti-apoptosis [175]	[174,175]

Abbreviations: SYB, Safflor yellow B; I/R, Ischemia/Reperfusion; HIE, Hypoxia–Ischemia Encephalopathy; HFD, High-fat diet; AD, Alzheimer’s Disease; TMF, 5,6,7,4′-Tetramethoxyflavanone; SCI, Spinal Cord Injury; AMP, Ampelopsin; TMP, Tetramethylpyrazine; iNOS, inducible nitric oxide synthase; DHC, Dihydrocapsaicin; BBB, blood–brain barrier; Aβ, amyloid-β; OMT, Oxymatrine; 6-OAP, 6-O-angeloylplenolin; LPS, Lipopolysaccharide; AS, Astragalus membranaceus saponins; GS-Rb1/3, Ginsenoside Rb1/3; MS, Multiple Sclerosis; MG, Mulberrofuran G; ER, endoplasmic reticulum; VB-1, Vitexin compound B-1; MFA, Methyl ferulic acid; EGCG, Epigallocatechin-3-gallate; TMQ, Thymoquinone; TBI, Traumatic Brain Injury; SD, Sleep Deprivation; SFN, Sulforaphane; CSDS, chronic social defeat stress.

**Table 2:** Summary of the features of different therapeutic strategies.

Category	Therapeutic Strategy	Features	References
Gene Therapy	Gene silencing (siRNA/shRNA)	Downregulation of NOX4 gene expression	[177,178,179,180,181,182]
Nanodrug Delivery Systems (NDDSs)	Liposomes/Polymeric nanoparticles	Surface modification with ligands for drug delivery across the BBB	[183,184]
	Biomimetic nanocarriers	Integration of innate cell membrane properties	[109,185,186]
	Exosomes	Cell-secreted endogenous nanoscale vesicles	[42,187,188]
	Nanozymes, gold nanoparticles, and black phosphorus nanosheets	Mimicry of natural antioxidant enzyme activity and function	[189,190,191]

Abbreviations: siRNA, small interfering RNA; shRNA, short hairpin RNA; BBB, blood–brain barrier; NDDSs, nanodrug delivery systems.

## Data Availability

No new data were created or analyzed in this study. Data sharing is not applicable to this article.

## Abbreviations

The following abbreviations are used in this manuscript:

α-Syn	α-synuclein
6-OAP	6-O-angeloylplenolin
AAVs	Adeno-associated viruses
AD	Alzheimer’s disease
ALP	Autophagy-lysosomal pathway
ALS	Amyotrophic Lateral Sclerosis
AMP	Ampelopsin
ARE	Antioxidant Response Element
AS	Astragalus membranaceus saponins
ATF3	Activating transcription factor 3
ATP	Adenosine triphosphate
Aβ	Amyloid-β
BBB	Blood–brain barrier
CNS	Central nervous system
CSDS	Chronic social defeat stress
DA	Dopaminergic
DBS	Deep brain stimulation
DH	Dehydrogenase
DHC	Dihydrocapsaicin
DMTs	Disease-modifying therapies
DPI	Diphenylene iodonium
Drp1	Dynamin-related protein 1
E-loop	Extracellular loop
ECHS1	Short-chain enoyl-CoA hydratase 1
EF-hands	N-terminal calcium-binding domains
EGCG	Epigallocatechin-3-gallate
ER	Endoplasmic reticulum
ETC	Electron transport chain
FAD	Flavin adenine dinucleotide
Fe^2+^	Ferrous iron
GABAergic	γ-aminobutyric acid-ergic
GPX4	Glutathione peroxidase 4
GS-Rb1/3	Ginsenoside Rb1/3
H_2_O_2_	Hydrogen peroxide
HFD	High-fat diet
HIE	Hypoxia–ischemia encephalopathy
I/R	Ischemia/Reperfusion
iNOS	inducible nitric oxide synthase
L-Dopa	Levodopa
LCN2	Lipocalin-2
LID	L-Dopa-induced dyskinesia
LPS	Lipopolysaccharide
MFA	Methyl ferulic acid
MG	Mulberrofuran G
miRNAs	MicroRNAs
MMP	Mitochondrial membrane potential
MS	Multiple Sclerosis
mtROS	Mitochondrial ROS
NADPH	Nicotinamide adenine dinucleotide phosphate
NDDs	Neurodegenerative diseases
NDDSs	Nanodrug delivery systems
NLRP3	NOD-like receptor thermal protein domain associated protein 3
NO	Nitric oxide
NOS	Nitric oxide synthase
NOX	NADPH oxidase
NP	Nanoparticles
Nrf2	Nuclear factor-erythroid 2 related factor 2
O_2_^•−^	Superoxide anion
OMT	Oxymatrine
PD	Parkinson’s disease
PHD	Peroxidase-homology domain
PKCα	Protein kinase Cα
RISC	RNA-induced silencing complex
ROS	Reactive oxygen species
SCI	Spinal Cord Injury
SD	Sleep Deprivation
SFN	Sulforaphane
shRNA	Short hairpin RNA
siRNA	Small interfering RNA
SNpc	Substantia nigra pars compacta
SOD1	Superoxide dismutase 1
SYB	Safflor yellow B
TBI	Traumatic Brain Injury
TFRC	Transferrin receptors
TM	Transmembrane
TMF	5,6,7,4′-Tetramethoxyflavanone
TMP	Tetramethylpyrazine
TMQ	Thymoquinone
UPS	Ubiquitin-proteasome system
VB-1	Vitexin compound B-1
XO	Xanthine oxidase
